# Knowledge, attitudes, and behaviours related to reduced-sodium salt: a systematic review

**DOI:** 10.1038/s41371-025-01098-2

**Published:** 2025-11-27

**Authors:** Katrina R. Kissock, Nadine Ghammachi, Annet C. Hoek, James D. Bullen, Jacqui Webster, Simone Pettigrew, Nitika Garg, Bruce Neal, Kathy Trieu

**Affiliations:** 1 The George Institute for Global Health, University of New South Wales, Sydney, NSW Australia; 2 School of Public Health, University of Technology Sydney, Sydney, NSW Australia; 3 Business School, University of New South Wales, Sydney, NSW Australia; 4 School of Public Health, Imperial College London, London, UK

**Keywords:** Risk factors, Hypertension

## Abstract

Despite consistent evidence of the cardioprotective benefits of reduced-sodium salt, it remains an underutilised intervention. Understanding how reduced-sodium salt is perceived is required to scale-up its use. This review summarises end user and healthcare professional knowledge, attitudes, and behaviours about reduced-sodium salt. We systematically searched four databases (inception to February 2024) and identified studies reporting knowledge, attitudes and behaviours towards reduced-sodium salt. Twenty-nine studies from 11 countries were included, 18 of which were intervention studies involving reduced-sodium salt and 11 were descriptive studies examining perceptions among the general community and healthcare professionals. Among intervention studies, there was high overall acceptability but mixed findings on taste. Outcomes related to use or willingness to use were mostly positive especially following cost-subsidisation. Among descriptive studies, there was low awareness (ranging from 0–32%) and reported use (10–16%) of reduced-sodium salt among the general community. Barriers to use included low availability and higher costs compared to regular salt. Awareness was higher among healthcare professionals (71%). Overall, most studies found high acceptability and willingness to use following exposure to reduced-sodium salt, despite some detecting taste differences. Greater awareness coupled with strategies to improve availability and affordability are important to scale-up the use of reduced-sodium salt.

## Introduction

Sodium is an essential dietary component for normal physiological functioning [1]. However, excess sodium consumption, usually in the form of salt (sodium chloride), is a major risk factor for noncommunicable diseases [2]. A diet high in sodium causes raised blood pressure and kidney disease, leading to millions of premature strokes, heart attacks, and deaths globally each year [3–5]. Currently, average global sodium intake is double that recommended by the World Health Organization (WHO) [6, 7] and estimated to be about 10 times more than that likely consumed by our hominid ancestors during a million years of evolution [1]. Decreasing sodium intake has been shown to reduce blood pressure in both children and adults [8, 9], and reduce the risk of cardiovascular diseases [9], with potential for substantial savings in healthcare costs [10]. However, sodium-reduction strategies implemented across the globe so far have largely been unsuccessful in meaningfully reducing average population sodium consumption [11]. Currently, no country is on track to meet the WHO 2025 goal of reducing sodium intake by 30% [12].

An underutilised intervention for reducing population sodium intake is the replacement of regular salt (sodium chloride) with reduced-sodium salt, either by consumers or the food processing industry. Reduced-sodium salt has a proportion of the sodium chloride replaced with a non-sodium component, most frequently potassium chloride but also sometimes magnesium chloride, calcium chloride, or magnesium phosphate [13]. There is now compelling evidence to show that substitution of regular salt with reduced-sodium salt lowers blood pressure among diverse populations and reduces the risk of stroke and cardiovascular mortality [13, 14]. The next step is to scale up global use of reduced-sodium salt in place of regular salt by ensuring that there is adequate consumer acceptability and demand [15]. To understand what interventions are needed to scale-up the use of reduced-sodium salt, it is important to examine current end user and healthcare professional knowledge, attitudes, and behaviours (KAB) related to reduced-sodium salt. In 2014, 45 countries had population-level data on salt-related KAB to inform national sodium-reduction strategies [16], however KABs related to reduced-sodium salt in particular are unknown. Therefore, this study systematically reviewed end user and healthcare professional KABs of reduced-sodium salt.

## Materials and methods

This review was registered in the International Prospective Register of Systematic Reviews (PROSPERO) in February 2023 under the registration number (CRD42023398163). We adhered to the Preferred Reporting Items for Systematic Reviews and Meta-Analyses (PRISMA) 2020 guidelines (Supplementary Table 1).

### Search strategy

A systematic search of peer-reviewed and grey literature published up to 8 February 2024 was conducted, which included an initial search in February 2023 and updated search in February 2024. Three electronic databases including PubMed, Cumulative Index to Nursing and Allied Health Literature (CINAHL) Plus, and Cochrane Central Register for Controlled Trials (CENTRAL) were systematically searched. An additional Google Scholar search was performed with the first 10 pages (100 results) reviewed for both the initial and updated searches. A combination of keywords and subject headings (including Medical Subject Headings [MeSH]) were used following the PICOS (population, intervention, comparison, outcome, setting/design) approach including salt substitute, reduced sodium salt, low sodium salt, potassium salt, consumer, user, knowledge, perception, behaviour, barrier, enabler, attitude, awareness, belief, and associated synonyms (Table 1). A detailed example of the search strategy is shown in Supplementary Table 2. Reference lists of relevant systematic reviews were also searched, and references identified by experts in the field were included.

**Table 1:** PICOS criteria used to define research questions.

Parameter	Description
Population	End users (consumers or chefs) or healthcare professionals
Intervention	Intake or exposure to reduced-sodium salt where some or all the sodium chloride in salt is replaced with a non-sodium salt component such as potassium chloride, magnesium chloride, magnesium phosphate, or calcium chloride.
Comparison	No comparator or a comparison to regular salt (100% NaCl)
Outcomes	Quantitative or qualitative measures of knowledge, attitudes, or behaviours related to reduced-sodium salt
Setting/design	Cohort, cross-sectional, randomised-controlled intervention or non-randomised intervention in humans

### Selection criteria

All studies reporting quantitative or qualitative outcome measures of knowledge, attitudes, and behaviours related to reduced-sodium salt were included. For the purpose of this review, we classified outcomes of interest based on previously explored salt related KAB outcomes [17] as listed in Table 2 including characteristics related to overall acceptability (defined as overall acceptability or liking of the product [18]) and current behaviours around replacing regular salt with reduced-sodium salt. Willingness to use reduced-sodium salt was classified as a behaviour outcome due to the recognition of intent as a proxy for behaviour [19]. In this review, reduced-sodium salt refers where some or all the sodium chloride in salt was replaced with a non-sodium salt component such as potassium chloride, magnesium chloride, magnesium phosphate, or calcium chloride. Studies where reduced-sodium salt was added during the manufacturing process of foods were excluded because it was covered in another review [18] and product-specific KABs are not generalisable to the entire food supply (e.g. the acceptability of processed meats that contain reduced-sodium salt differs from that of breads that contain reduced-sodium salt). Studies could include any population of users (including general community and intervention study participants (collectively termed consumers), chefs, and healthcare professionals) of any age or gender, and with or without pre-existing health conditions. To ensure only KABs from the general population were obtained, studies with trained judges or panellists, namely those individuals who are trained to conduct taste tests in research and development and therefore have a higher ability to detect taste differences compared to the general population, were excluded. Cohort studies, cross-sectional studies, and intervention studies were included with duplicate reports of the same data removed. In intervention studies, only baseline knowledge of reduced-sodium salt was included, while outcomes related to attitudes and behaviours were included at both baseline and follow-up. Studies not published in English were also excluded.

**Table 2:** Examples of knowledge, attitude and behaviour outcomes of interest.

	Outcome
**Knowledge**	Awareness of reduced-sodium salt
	Awareness of the health impacts of reduced-sodium salt
	Awareness of recommendations by healthcare professionals for use of reduced-sodium salt in people with various health conditions
**Attitude**	Perceived taste
	Overall acceptability/liking
	Preference
	Accessibility (including availability and affordability)
	Methods to recommend reduced-sodium salt and by whom
**Behaviour**	Use of reduced-sodium salt by end users and healthcare professionals
	Willingness to use reduced-sodium salt
	Prescription or recommended use of reduced-sodium salt in clinical practice by healthcare professionals

### Study selection

The title-abstract and full-text screening was conducted by two researchers independently (KRK and NG). Discrepancies at each screening stage were discussed and consensus was reached through consultation with a third researcher (KT).

### Data extraction and analysis

Data from relevant studies were extracted by a single researcher (NG) and checked by a second researcher (KRK). The following data were extracted into an Excel spreadsheet: article citation, country, study setting, study type (intervention or descriptive), study design (cross-sectional, randomised-controlled intervention, non-randomised intervention), name of study cohort (if applicable), sample size, participant type (general population or healthcare professionals), mean participant age and sex, outcomes of interest (knowledge, attitude, and behaviour results) including p-values (where applicable), and method to assess the outcome of interest. Information about the reduced-sodium salt intervention (i.e. how the reduced-sodium salt was provided), composition of reduced-sodium salt used, and comparator salt (if applicable) were also extracted for intervention studies. Two narrative syntheses were conducted, one for intervention studies (studies evaluating knowledge, attitudes, or behaviours related to reduced-sodium salt following an intervention) and one for descriptive studies (cross-sectional and baseline studies). Results from each outcome in each study were categorised as low (0–29%), moderate or mixed (30–49%), or high (≥50%) for knowledge and behaviour-related outcomes. All attitude-related outcomes and outcomes related to willingness to use were categorised as negative, neutral/mixed, or positive based on how each study reported their findings. Due to large heterogeneity between study methodologies and outcomes reported, a meta-analysis was not feasible.

### Quality assessment

The quality assessment of included articles was conducted by two researchers independently (KRK and NG) and discrepancies were discussed between researchers to reach final consensus. Quality was assessed using the revised Cochrane Risk of Bias tool (RoB 2.0) [20] for randomised-controlled intervention studies. For non-randomised intervention studies and cross-sectional descriptive studies, the Newcastle-Ottawa Scale (NOS) was used with adaptations made based on the original NOS [21] and the adaptation by Herzog et al. (2013) for both types of studies (see Supplementary Material 1 for further details). The type of risk of bias tool used was determined based on the relevant outcome of interest. Process evaluations of consumer interventions were assessed as a cross-sectional study. Randomised-controlled studies could score “low risk of bias”, “some concerns”, or “high risk of bias” for overall study quality. Maximums of 10 and 11 stars could be achieved for cross-sectional studies and non-randomised intervention studies, respectively. Studies obtaining the maximum number of stars (10 or 11 depending on the study design) were considered of highest quality, whereas studies obtaining no stars were considered of lowest quality. Studies were not excluded from the review if they scored “high risk of bias” or were of lowest quality as we aimed to comprehensively summarise all available evidence to provide full contextual and global understanding. Excluding studies based on bias or quality may have limited the inclusion of data of geographical diversity and some outcomes of interest.

## Results

### Study characteristics

**Fig. 1:**
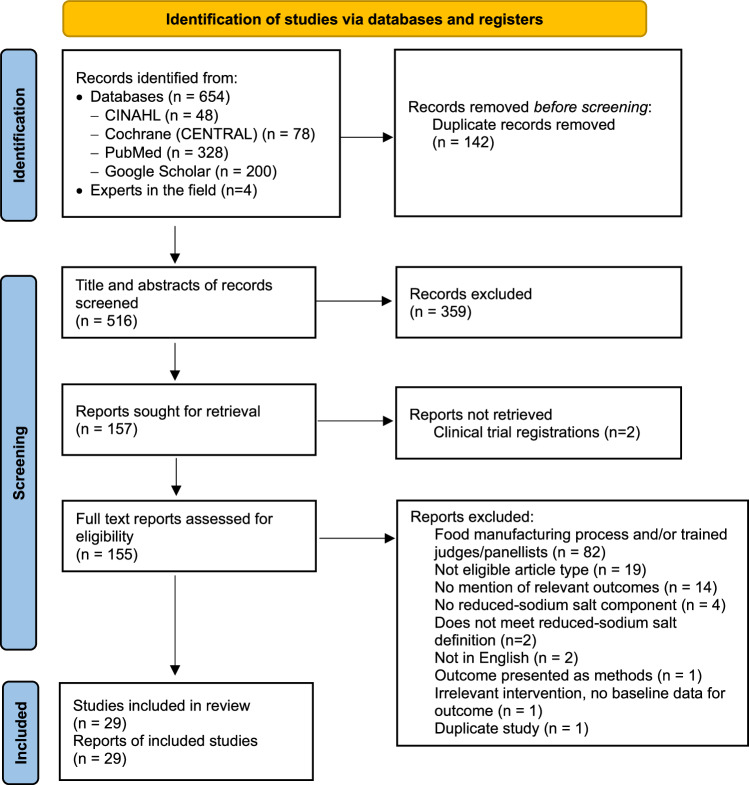
PRISMA flow diagram for systematic review study selection.

Initially, 658 articles were identified, with 516 articles screened by title and abstract after the removal of duplicates. Following full-text assessment of 155 articles, 29 studies were eligible and included in the review. Primary reasons for exclusion at full-text assessment were that studies related to the food manufacturing process and/or involved trained judges/panellists, there was no mention of KAB outcomes related to reduced-sodium salt, or articles were not an eligible type (conference abstract, data brief, review, or study protocol) (Fig. 1).

Of the 29 articles included, 18 were intervention studies that assessed KABs following an intervention involving reduced-sodium salt (11 randomised and seven non-randomised trials) and 12 were descriptive studies assessing KABs without a reduced-sodium salt intervention (Supplementary Table 3 & 4). One intervention study [22] reported outcomes of interest at both baseline and follow-up, where results were synthesised as both a descriptive and an intervention study respectively. Most studies were conducted in China (31%) and the United States (24%), at a local community level among general consumers, including both male and female participants, with a mean participant age range of 38–70 years. The characteristics of included studies are summarised in Table 3. Twenty-three studies reported quantitative outcomes, three reported qualitative outcomes, and three reported a combination of quantitative and qualitative outcomes. Approximately half (n = 14) of all studies reported more than one outcome of interest related to knowledge, attitudes, and behaviour, while the other half (n = 15) reported only a single outcome of interest, with attitude being the most commonly reported outcome. The average number of relevant outcomes per study was 1.7 across all studies and 2.5 in studies with >1 outcome. Outcomes were mostly assessed using questionnaires (n = 21), with interviews (n = 5), focus groups (n = 3), visual analogue scales (n = 1), Likert scales (n = 1), and unknown methods of self-reporting (n = 2) also used.

**Table 3:** Summary of characteristics for included studies.

	All studies (n = 29)^a^	Intervention studies (n = 18)	Descriptive studies (n = 12)
	n		
Country			
China	9	4	5
United States	7	3	4
India	3	1	3
South Africa	3	3	0
Other^b^	7	7	0
Sex			
Both male and female	23	15	9
Female only	1	1	0
Did not report	5	2	3
Participant type			
Consumers/patients	24	18	7
Healthcare professionals^c^	5	0	5
Age range^d^			
Adults (≥18 years)	20	15	6
Children and adults	1	1	0
Not specified	3	2	1
Setting			
Community level	17	11	6
Population level (national or provincial)	12	7	6
Knowledge outcomes			
Awareness of reduced-sodium salt	5	0	5
Awareness of recommendations for use in people with various health conditions	5	0	5
Awareness of health impacts	1	0	1
Attitude outcomes			
Perceived taste	15	14	1
Overall acceptability	5	5	0
Preference	3	3	0
Accessibility (availability and affordability)	3	2	1
Recommendation (methods and by whom)	1	0	1
Behaviour outcomes			
Use of reduced-sodium salt	8	3	5
Willingness to use reduced-sodium salt	4	4	0
Prescription of reduced-sodium salt in practice	1	0	1

^a^One study included as both an intervention study and a descriptive study.

^b^Other countries included: Australia (n = 1), Brazil (n = 1), Finland (n = 1), Iran (n = 1), New Zealand (n = 1), Peru (n = 1), and Taiwan (n = 1).

^c^Healthcare professional studies included nurses (n = 4) and doctors (n = 1).

^d^Studies conducted in consumers/patients only (n = 24). Excludes studies conducted in healthcare professionals.

### Intervention studies of reduced-sodium salt

**Fig. 2:**
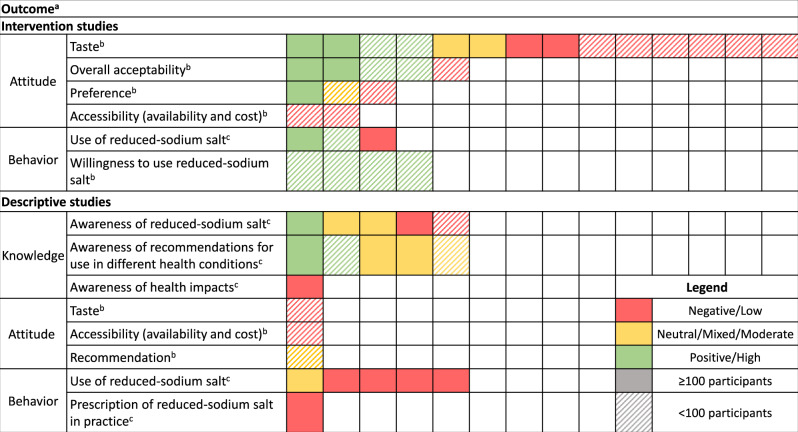
Summary of knowledge, attitude, and behaviour outcomes across intervention and descriptive studies. a Each coloured box represents an individual study within each outcome, for example there are 14 individual intervention studies reporting on taste outcomes. b Attitudes and willingness to use were classified as red if negative outcomes were reported, yellow if neutral or mixed outcomes were reported, and green if positive outcomes were reported. c Rates of knowledge, use or prescription were classified as low (red) between 0–29%, moderate or mixed (yellow) between 30–49%, and high (green) when ≥ 50%.

Eighteen studies evaluated consumer and/or patient perceptions following an intervention involving reduced-sodium salt in 11 countries, mostly at a local community level (Table 3 and Supplementary Table 3). The interventions included providing the reduced-sodium salt directly to consumers for testing by itself (without food) (n = 2) [23, 24], with crackers (n = 1) [25], or in a water solution (n = 1) [26]; providing the salt directly to end users for use in the home (n = 10) or in food outlets (n = 1) [27]; making the salt available for immediate use or to take home free of charge at various locations (n = 1) [28]; and making the salt available for purchase in grocery stores (n = 2) [29, 30]. Of these studies, five provided parallel education on sodium reduction, [28–32] one on hypertension [33] and one on reduced-sodium salt [27]. One study explored the effect of a price subsidy [30]. The composition of reduced-sodium salt varied widely across studies, ranging from 100% potassium chloride (n = 1) [34] to various blends of i) sodium chloride and potassium chloride (n = 5) [22, 23, 28, 32, 35]; ii) sodium chloride, potassium chloride, and magnesium salts (including magnesium sulphate and magnesium chloride) (n = 4) [36–39]; and iii) potassium, calcium, and/or magnesium and/or phosphate-based salts (n = 1) [24]. Follow-up for outcomes of interest were identified in 12 studies, ranging from four weeks to three years, while four studies did not have a follow-up period. Among intervention studies, findings were largely positive in terms of overall acceptability, use, and willingness to use reduced-sodium salt. Findings for taste were mixed in larger studies with ≥100 participants, while negative outcomes were mostly reported in smaller studies with <100 participants (Fig. 2).

### Attitude outcomes in intervention studies

Of the five studies assessing overall acceptability after tasting reduced-sodium salt, four reported high acceptability with no or little difference to regular salt among consumers in China [35], Iran [23], Peru [27], and South Africa [33], while there was low acceptance among participants in Brazil [32] (Supplementary Table 5). The studies conducted in China, Iran, and Brazil blinded participants to the reduced-sodium salt intervention. The study in Iran reported more participants preferred the reduced-sodium salt (containing up to 30% potassium chloride) compared to regular salt, [23] while one study in the United States reported most participants preferred taking a potassium supplement over a 100% potassium chloride-based salt [34]. Finally, studies in China reported consumers identified limited accessibility to reduced-sodium salt due to low availability and higher costs compared to regular salt as barriers to use [29, 35].

Fourteen studies specifically focusing on the perceived taste of the provided reduced-sodium salt had mixed findings. In four studies (n = 1 224) in China, India, and New Zealand, the majority of consumers reported positive or little to no taste differences [22, 31, 35, 37]. In contrast, eight studies (n = 609) reported negative findings related to perceived taste, with some identifying reduced-sodium salt as tasting less salty than regular salt [26, 27, 29, 39] or having an unfavourable taste (e.g. bitter, chemical, metallic, or strange taste) [26, 32, 38, 39]. In most of these studies, the reduced-sodium salt had >35% of the sodium chloride replaced with a non-sodium component. Two studies (n = 84) reported mixed taste outcomes for various reduced-sodium salts [24, 25] (Supplementary Table 6).

### Behaviour outcomes in intervention studies

Use of reduced-sodium salt, measured through self-reported use as yes/no responses, was high (53–82%) among two intervention studies in China [29, 30] and low (26%) in one intervention study in Australia [28]. One of the studies in China found that subsidizing the cost of reduced-sodium salt increased the use among consumers up to 83% compared to 50% when not subsidized [30]. Willingness to continue use of reduced-sodium salt was positive in all four intervention studies reporting the outcome. Three studies conducted in China, Peru and South Africa (using a 25% potassium-chloride-based salt [27, 35] or salt of unknown composition [33]) reported most consumers were willing to continue using the reduced-sodium salt following completion of the intervention. In another study conducted in South Africa, a higher proportion of consumers were willing to use a 50% potassium chloride-based reduced-sodium salt compared to salts with 35, 66, and 100% potassium chloride [25].

### Descriptive studies of end user and healthcare professional perceptions of reduced-sodium salt

Twelve cross-sectional or baseline analyses examined perceptions of reduced-sodium salt among consumers or healthcare professionals without exposure to a reduced-sodium salt intervention (Supplementary Table 4). Seven were conducted among consumers at the population level in China (n = 5) and India (n = 2) and five among healthcare professionals at the local community level in the United States (n = 4) and India (n = 1) (Table 3). Most descriptive studies had ≥100 participants and reported moderate levels of knowledge and low levels of behaviour outcomes. Attitude-related outcomes were mixed or negative and assessed in studies with <100 participants (Fig. 2).

### Knowledge outcomes in descriptive studies

The awareness around reduced-sodium salt varied between 0–71% across five studies conducted at the population level in China and India. High rates of awareness of reduced-sodium salt were found among doctors in India (71% of 165 participants) [40]. There was no awareness by consumers in India (0% of 502 participants) [22], and low to moderate awareness by consumers in Zhejiang Province in China (30% of 7512 participants) [41], across six provinces in China (32% of 4 000 participants) [42], and among consumers and influencers (which included healthcare professionals, members of women self-help groups, village heads, and members of resident welfare associations) in India [43]. In Zhejiang Province China, 21% of consumers were aware that reduced-sodium salt helps control blood pressure [41]. Four studies conducted among nurses in the US (n = 15 to 300) found 41 to 66% correctly identified that using reduced-sodium salt to season food should not be recommended to patients with heart failure [44–47]. Similarly, 44% of doctors in Mangalore, India did not know the exact indication for the use of reduced-sodium salt, while 53% reported that it is used in hypertension [40]. In the same group of doctors, 72% did not know about contraindications to the use of reduced-sodium salt.

### Attitude outcomes in descriptive studies

Only one descriptive study reported attitudes towards reduced-sodium salt [43]. In this study, consumers and healthcare professionals in India reported barriers related to the lack of availability and higher cost of reduced-sodium salt compared to regular salt. Availability and affordability were key factors in their purchase decision, and participants identified that a subsidy would be beneficial to offset the extra cost of the reduced-sodium salt. Consumers also identified that healthcare professionals are the most trusted source for promoting and recommending use of reduced-sodium salt.

### Behaviour outcomes in descriptive studies

In five descriptive studies, the regular use of reduced-sodium salt by consumers in China (captured as yes/no responses) was found to be low to moderate, ranging between 10 to 16% among the general population (sample sizes ranged from 4 000 to 179 834) [41, 42, 48, 49] and up to 43% among consumers who were following the public platform of China Healthy Lifestyle for All Campaign on WeChat (and thus more concerned about diet and health, study n = 12,732) [50]. Among doctors in Mangalore, India, 19% (of 165 participants) reported prescribing reduced-sodium salt to patients in clinical practice [40].

### Quality assessment

Following assessment using modified NOS tools, study quality among cross-sectional descriptive studies ranged from three to eight stars out of a possible 10 stars, with the majority (n = 8) of moderate quality (4–6 stars) and two studies of high quality (7–10 stars) (Supplementary Table 7, 8). Study quality among non-randomised evaluation studies ranged from 3–5 stars out of a possible 11 stars, with four studies of lower quality (1–4 stars) and two studies of moderate quality (5–7 stars) (Supplementary Table 9, 10). Most studies were scored as lower quality because sample size was not justified, the study did not control for age or sex, and/or no statistical tests were completed. Following assessment using the Cochrane Risk of Bias 2.0 tool, study quality among randomised-controlled evaluation studies was mostly classified as “high risk of bias” (n = 4) or “moderate risk of bias” (n = 4), while one was “low risk of bias” (Supplementary Table 11).

## Discussion

We found that despite some negative findings related to taste, reduced-sodium salt was considered overall acceptable and there were high levels of a willingness to use following exposure to reduced-sodium salt. Among general consumers, awareness of reduced-sodium salt was moderate, while the use was mostly low. However, there was increased acceptability and use of reduced-sodium salt when addressing barriers related to accessibility, affordability and availability by making it widely available and providing price subsidises. In contrast, healthcare professionals had high levels of awareness.

Overall acceptability of different compositions of reduced-sodium salt compared to regular salt was high across countries. However, there were mixed findings regarding taste, with about half of studies identifying a different or unfavourable taste of reduced-sodium salt compared to regular salt. Unfavourable taste described as bitter and less salty compared to regular salt was reported mostly in smaller studies with <100 participants and when >35% of the sodium chloride was replaced with a non-sodium component (e.g. potassium chloride or magnesium phosphate). In large population-based studies, there were formulations that resulted in taste being considered positive by most people, notably at 25–30% potassium chloride replacement. Taste is very subjective and variable such that assessment amongst large numbers of people is often required to increase reliability of results [51]. It is also important to note that detecting taste differences should not be confused with rejection [26]. Taste constitutes only one aspect of overall acceptability, which also considers other sensory aspects, familiarity, ease of use, and consumer expectations [52]. So, while participants may detect taste differences compared to regular salt, the salt may still be overall acceptable. It is recommended that along with testing different reduced-sodium salt mixtures, future studies should measure consumer acceptability over longer exposure times to assess whether consumers adapt to taste differences and thus have increased acceptability of the product [26, 28]. In this review, most studies adopted shorter exposure periods. There is also a need to update and synthesise the evidence about perceptions and acceptability of packaged and processed foods containing reduced-sodium salt among the general population. This is important as packaged and processed foods are the main sources of dietary sodium intake in high-income and Western countries, and growing contributors in low- and middle-income countries [17].

Our review found that healthcare professionals in India and the US had moderate to high rates of awareness and knowledge around the suitability of reduced-sodium salt for different patient groups. However, in the study in India, the prescription or recommendation rate of reduced-sodium salt was low. Increasing healthcare professionals’ knowledge and mobilising them to recommend reduced-sodium salt to people who can benefit is crucial for scale-up. This approach not only helps accelerate the switch among hypertensive patients who can benefit most from reduced-sodium salt [53, 54], but helps mitigate concerns about the use of potassium-enriched, reduced-sodium salt among people with heart failure and advanced chronic kidney disease who may be at increased risk of hyperkalaemia [55, 56]. This could be combined with other measures that may help minimise risks among certain population groups at risk of hyperkalaemia, such as the display of warning labels and education [57, 58].

Our systematic review found limited data about consumers’ KAB related to reduced-sodium salt, with most surveys including only one or two questions. More data are required, particularly in low- and middle-income countries where discretionary salt use is the main source of dietary sodium intake compared to salt within packaged foods [59]. KAB questions related to reduced-sodium salt should be integrated into future surveys and standardised questionnaires assessing salt-related KAB. This includes the WHO STEPS NCD risk factor surveillance survey that currently includes 12 KAB questions related to salt and is regularly conducted in many low- and middle-income countries [60]. Understanding consumers’ perceptions of reduced-sodium salt is critical to inform the development of strategies to reduce population sodium intake and associated disease burden.

### Strengths & Limitations

This review is the first, to our knowledge, to summarise the literature relating to end user and healthcare professional perceptions of reduced-sodium salt. The findings are important, especially with growing interest in promoting reduced-sodium salt as an effective global strategy for population sodium reduction leading to decreased blood pressure and the risk of stroke, cardiovascular disease and premature death. Strengths of this review include exploration of reduced-sodium salt perceptions across diverse countries, the comprehensive search strategy, and search for peer reviewed and grey literature. The primary limitation was the difficulty in formally comparing study findings due to the heterogeneity of studies in terms of outcomes measured and composition of reduced-sodium salt. Most studies were also conducted in China or the United States, thus these findings may not be generalisable to other population groups. Another important limitation is that outcomes of interest in the current review were mostly secondary or exploratory outcomes where statistical tests were not performed and limited detail around the outcome was provided. Many studies assessing attitude-related outcomes had small sample sizes and may affect reliability of results. Furthermore, there were limited studies involving healthcare professionals and no studies were conducted with chefs or other types of users, limiting the scope of the findings. In terms of healthcare professionals, only one study was conducted with doctors, while four were conducted with nurses or nursing students with a focus on heart failure patients, also limiting the generalisability of results.

This review finds that overall acceptability and use or willingness to use among end users exposed to a reduced-sodium salt intervention were high in most studies, despite some studies observing taste differences. Surveys revealed no to moderate levels of awareness and low use of reduced-sodium salt among the general population in India and China, with low availability and high cost commonly cited barriers to the uptake of reduced-sodium salt. Increasing availability and price subsidies of reduced-sodium salt were shown to increase usage and should be considered as part of strategies to support future scale-up. There is also a need for comprehensive and standardised measurement and monitoring of knowledge, attitudes, and behaviours related to reduced-sodium salt to inform and evaluate implementation globally.

## Summary

### What is known about the topic

- Replacing regular salt for reduced-sodium salt lowers blood pressure among diverse populations and reduces the risk of stroke and cardiovascular mortality.
- Numerous studies evaluate aspects of knowledge, attitudes or behaviours related to reduced-sodium salt, but broader understanding is lacking. Understanding how reduced-sodium salt is perceived is required to scale-up its use.

### What this study adds

- This study provides a comprehensive overview of end user and healthcare professional knowledge, attitudes and behaviours about reduced-sodium salt.
- Our study shows high acceptability and a willingness to use reduced-sodium salt despite some detecting taste differences.
- Greater awareness coupled with strategies to improve availability and affordability are important to scale-up the use of reduced-sodium salt.

## Supplementary information

Supplementary Material

## Data Availability

The datasets generated and analysed during the current study are available from the corresponding author on reasonable request.

## References

[1] Jaques DA, Ponte B. Dietary sodium and human health. Nutrients. 2023;15:3696. 37686728 10.3390/nu15173696 PMC10490134

[2] Whelton PK. Sodium, blood pressure, and cardiovascular disease. Circulation. 2014;129:1085–7. 24425752 10.1161/CIRCULATIONAHA.114.008138

[3] GBD 2017 Diet Collaborators. Health effects of dietary risks in 195 countries, 1990-2017: A systematic analysis for the Global Burden of Disease Study 2017. Lancet. 2019;393:1958–72. 30954305 10.1016/S0140-6736(19)30041-8 PMC6899507

[4] Wang L, Du J, Cao W, Sun S. Trends of stroke attributable to high sodium intake at the global, regional, and national levels from 1990 to 2019: a population-based study. Neurol Res. 2021;43:474–81. 33377423 10.1080/01616412.2020.1867950

[5] Ma Y, He FJ, Sun Q, Yuan C, Kieneker LM, Curhan GC, et al. 24-hour urinary sodium and potassium excretion and cardiovascular risk. N Engl J Med. 2022;386:252–63. 34767706 10.1056/NEJMoa2109794 PMC9153854

[6] Powles J, Fahimi S, Micha R, Khatibzadeh S, Shi P, Ezzati M, et al. Global, regional and national sodium intakes in 1990 and 2010: a systematic analysis of 24 h urinary sodium excretion and dietary surveys worldwide. BMJ Open. 2013;3:e003733. 24366578 10.1136/bmjopen-2013-003733 PMC3884590

[7] World Health Organization. Guideline: Sodium intake for adults and children. Geneva, World Health Organization; 2012. 23658998

[8] He FJ, Li J, MacGregor GA. Effect of longer term modest salt reduction on blood pressure: Cochrane systematic review and meta-analysis of randomised trials. BMJ. 2013;346:f1325. 23558162 10.1136/bmj.f1325

[9] Aburto NJ, Ziolkovska A, Hooper L, Elliott P, Cappuccio FP, Meerpohl JJ. Effect of lower sodium intake on health: Systematic review and meta-analyses. BMJ. 2013;346:f1326. 23558163 10.1136/bmj.f1326 PMC4816261

[10] Smith-Spangler CM, Juusola JL, Enns EA, Owens DK, Garber AM. Population strategies to decrease sodium intake and the burden of cardiovascular disease. Ann Intern Med. 2010;152:481–7. 20194225 10.7326/0003-4819-152-8-201004200-00212

[11] He FJ, Brown M, Tan M, MacGregor GA. Reducing population salt intake - An update on latest evidence and global action. J Clin Hypertens. 2019;21:1596–601. 10.1111/jch.13664 PMC8030337 31448517

[12] Santos JA, Tekle D, Rosewarne E, Flexner N, Cobb L, Al-Jawaldeh A, et al. A systematic review of salt reduction initiatives around the world: A midterm evaluation of progress towards the 2025 global non-communicable diseases salt reduction target. Adv Nutr. 2021;12:1768–80. 33693460 10.1093/advances/nmab008 PMC8483946

[13] Brand A, Visser ME, Schoonees A, Naude CE. Replacing salt with low‐sodium salt substitutes (LSSS) for cardiovascular health in adults, children and pregnant women. Cochrane Database Syst Rev. 2022;8:CD015207. 35944931 10.1002/14651858.CD015207 PMC9363242

[14] Yin X, Rodgers A, Perkovic A, Huang L, Li K-C, Yu J, et al. Effects of salt substitutes on clinical outcomes: a systematic review and meta-analysis. Heart. 2022;108:1608–15. 35945000 10.1136/heartjnl-2022-321332

[15] Kissock KR, Garrett GS, Mkambula P, Bullen JD, Trieu K, Fisher LJ, et al. Switching the world’s salt supply—Learning from iodization to achieve potassium enrichment. Adv Nutr. 2024;15:100148. 37977326 10.1016/j.advnut.2023.100148 PMC10730351

[16] Trieu K, Neal B, Hawkes C, Dunford E, Campbell N, Rodriguez-Fernandez R, et al. Salt reduction initiatives around the world – A systematic review of progress towards the global target. PloS one. 2015;10:e0130247. 26201031 10.1371/journal.pone.0130247 PMC4511674

[17] McKenzie B, Santos JA, Trieu K, Thout SR, Johnson C, Arcand J, et al. The science of salt: A focused review on salt‐related knowledge, attitudes and behaviors, and gender differences. J Clin Hypertens. 2018;20:850–66. 10.1111/jch.13289 PMC8031068 29722131

[18] Jaenke R, Barzi F, McMahon E, Webster J, Brimblecombe J. Consumer acceptance of reformulated food products: A systematic review and meta-analysis of salt-reduced foods. Crit Rev Food Sci Nutr. 2017;57:3357–72. 26745848 10.1080/10408398.2015.1118009

[19] Ajzen I. Consumer attitudes and behavior: The theory of planned behavior applied to food consumption decisions. Riv Econ Agrar. 2015;70:121–38.

[20] Sterne JAC, Savović J, Page MJ, Elbers RG, Blencowe NS, Boutron I, et al. RoB 2: a revised tool for assessing risk of bias in randomised trials. BMJ. 2019;366:l4898. 31462531 10.1136/bmj.l4898

[21] Wells G, Shea B, O’Connell D, Peterson J, Welch V, Losos M, et al. The Newcastle-Ottawa Scale (NOS) for assessing the quality of nonrandomised studies in meta-analyses. Ottawa Hospital Research Institute, 2011. https://www.ohri.ca/programs/clinical_epidemiology/oxford.asp.

[22] Yu J, Thout SR, Li Q, Tian M, Marklund M, Arnott C, et al. Effects of a reduced-sodium added-potassium salt substitute on blood pressure in rural Indian hypertensive patients: A randomized, double-blind, controlled trial. Am J Clin Nutr. 2021;114:185–93. 33782684 10.1093/ajcn/nqab054

[23] Maleki A, Soltanian AR, Zeraati F, Sheikh V, Poorolajal J. The flavor and acceptability of six different potassium-enriched (sodium reduced) iodized salts: a single-blind, randomized, crossover design. Clin Hypertens. 2016;22:18. 28031983 10.1186/s40885-016-0054-9 PMC5178985

[24] Sopko JA. Freeman RM. Salt substitutes as a source of potassium. JAMA. 1977;238:608–10. 577961

[25] Crouch SH, Ware LJ, Norris SA, Schutte AE. Comparing a range of potassium-enriched low sodium salt substitutes to common salt: Results of taste and visual tests in South African adults. Nutr Metab Cardiovasc Dis. 2023;34:903–10. 38220506 10.1016/j.numecd.2023.12.015

[26] Sinopoli DA, Lawless HT. Taste properties of potassium chloride alone and in mixtures with sodium chloride using a check‐all‐that‐apply method. J Food Sci. 2012;77:S319–S22. 22901084 10.1111/j.1750-3841.2012.02862.x

[27] Lazo-Porras M, Del Valle A, Beran D, Pesantes MA, Perez-Leon S, Ponce-Lucero V, et al. Implementation of a salt substitute intervention using social marketing in resourced-limited communities in Peru: a process evaluation study. Front Public Health. 2023;11:1068624. 37275501 10.3389/fpubh.2023.1068624 PMC10235695

[28] Land MA, Wu JH, Selwyn A, Crino M, Woodward M, Chalmers J, et al. Effects of a community-based salt reduction program in a regional Australian population. BMC Public Health. 2016;16:388. 27169380 10.1186/s12889-016-3064-3 PMC4864903

[29] Chu H, Zhang J, Fetters MD, Niu W, Li H, Li N, et al. A mixed methods process evaluation of a clustered-randomized controlled trial to determine the effects of community-based dietary sodium reduction in rural China. Front Med. 2021;8:646576. 10.3389/fmed.2021.646576 PMC8192799 34124088

[30] Wang X, Li X, Vaartjes I, Neal B, Bots ML, Hoes AW, et al. Does education level affect the efficacy of a community based salt reduction program? - A post-hoc analysis of the China Rural Health Initiative Sodium Reduction Study (CRHI-SRS). BMC public health. 2016;16:759. 27515930 10.1186/s12889-016-3454-6 PMC4982434

[31] Eyles H, Grey J, Jiang Y, Umali E, McLean R, Te Morenga L, et al. Effectiveness of a sodium-reduction smartphone app and reduced-sodium salt to lower sodium intake in adults with hypertension: Findings from the salt alternatives randomized controlled trial. JMIR Mhealth Uhealth. 2023;11:e43675. 36892914 10.2196/43675 PMC10037177

[32] Barros CL, Sousa AL, Chinem BM, Rodrigues RB, Jardim TS, Carneiro SB, et al. Impact of light salt substitution for regular salt on blood pressure of hypertensive patients. Arq Bras Cardiol. 2015;104:128–35. 25409877 10.5935/abc.20140174 PMC4375656

[33] Lloyd-Sherlock P, Gómez-Olivé FX, Ngobeni S, Wagner RG, Tollman S. Pensions and low sodium salt: A qualitative evaluation of a new strategy for managing hypertension in Rural South Africa. Curr Aging Sci. 2018;11:140–6. 30019655 10.2174/1874609811666180718114250

[34] Hueston WJ. Use of salt substitutes in the treatment of diuretic-induced hypokalemia. J Fam Pract. 1989;29:623–6. 2687427

[35] Liu Y, Chu H, Peng K, Yin X, Huang L, Wu Y, et al. Factors associated with the use of a salt substitute in Rural China. JAMA Netw Open. 2021;4:e2137745. 34878549 10.1001/jamanetworkopen.2021.37745 PMC8655604

[36] Charlton KE, Steyn K, Levitt NS, Peer N, Jonathan D, Gogela T, et al. A food-based dietary strategy lowers blood pressure in a low socio-economic setting: A randomised study in South Africa. Public Health Nutr. 2008;11:1397–406. 18752692 10.1017/S136898000800342X

[37] Li N, Prescott J, Wu Y, Barzi F, Yu X, Zhao L, et al. The effects of a reduced-sodium, high-potassium salt substitute on food taste and acceptability in rural northern China. Br J Nutr. 2009;101:1088–93. 18710605 10.1017/S0007114508042360

[38] Pan W-H, Lai Y-H, Yeh W-T, Chen J-R, Jeng J-S, Bai C-H, et al. Intake of potassium- and magnesium-enriched salt improves functional outcome after stroke: A randomized, multicenter, double-blind controlled trial. Am J Clin Nutr. 2017;106:1267–73. 28877896 10.3945/ajcn.116.148536

[39] Pietinen P, Ruotsalainen P, Tanskanen A, Puska P. Sodium intake reduction in volunteer families by using a salt substitute and nutrition counselling. Ann Nutr Metab. 1981;25:371–80. 7332315 10.1159/000176518

[40] Fathima KA, Bhargava M. Salt reduction and low-sodium salt substitutes: Awareness among health-care providers in Mangalore, Karnataka. Indian J Community Med. 2018;43:266–9. 30662177 10.4103/ijcm.IJCM_22_18 PMC6319292

[41] Du X, Fang L, Xu J, Chen X, Bai Y, Wu J, et al. The association of knowledge, attitudes and behaviors related to salt with 24-h urinary sodium, potassium excretion and hypertensive status. Sci Rep. 2022;12:13901. 35974077 10.1038/s41598-022-18087-x PMC9381520

[42] Zhang P, Fan F, Li Y, Li Y, Luo R, Li L, et al. Awareness and use of low-sodium salt substitutes and its impact on 24-h urinary sodium and potassium excretion in China - A cross-sectional study. Nutrients. 2023;15:3000. 37447326 10.3390/nu15133000 PMC10346169

[43] Sehgal R, Srinivasapura Venkateshmurthy N, Khatkar R, Konkati SP, Jarhyan P, Sharma M, et al. Awareness and availability of low sodium iodized salt: Results from formative research of Promoting Uptake of Low SodiUm Iodized Salt by Rural and Urban HousehoLds in India-The PLURAL study. Nutrients. 2023;16:130. 38201960 10.3390/nu16010130 PMC10781031

[44] Albert NM, Collier S, Sumodi V, Wilkinson S, Hammel JP, Vopat L, et al. Nurses’ knowledge of heart failure education principles. Heart Lung. 2002;31:102–12. 11910385 10.1067/mhl.2002.122837

[45] Fowler S. Improving community health nurses’ knowledge of heart failure education principles: a descriptive study. Home Healthc Nurse. 2012;30:91–101. 22306754 10.1097/NHH.0b013e318242c5c7

[46] Washburn SC, Hornberger CA, Klutman A, Skinner L. Nurses’ knowledge of heart failure education topics as reported in a small midwestern community hospital. J Cardiovasc Nurs. 2005;20:215–20. 15870593 10.1097/00005082-200505000-00014

[47] Yehle KS, Chang K. The integration of an online module on student learning. Comput Inform Nurs. 2012;30:598–603. 22914215 10.1097/NXN.0b013e31825e1ed6

[48] Han B, Li C, Zhou Y, Zhang M, Zhao Y, Zhao T, et al. Association of salt-reduction knowledge and behaviors and Salt Intake in Chinese Population. Front Public Health. 2022;10:872299. 35509508 10.3389/fpubh.2022.872299 PMC9058069

[49] Zhang W, Neupane D, Zhao Z, Jiang B, Zhang M, Zhang X, et al. Knowledge and practices related to salt consumption in China: findings from a national representative cross-sectional survey. J Hum Hypertens. 2024;38:155–67. 10.1038/s41371-023-00861-7 PMC10844095 37857758

[50] Yang Y, Wang J, Ma J, Shi W, Wu J. Comparison of salt-related knowledge and behaviors status of WeChat Users between 2019 and 2020. Nutrients. 2021;13:2141. 34206633 10.3390/nu13072141 PMC8308297

[51] Gacula JrM, Rutenbeck S. Sample size in consumer test and descriptive analysis. J Sens Stud. 2006;21:129–45.

[52] Murray JM, Baxter IA Sensory evaluation - Food acceptability and sensory evaluation. In: Caballero B, editor. Encyclopedia of Food Sciences and Nutrition (Second Edition). Oxford: Academic Press; 2003. p. 5130-6.

[53] Bistola V, Arfaras-Melainis A, Trogkanis E, Bakosis G, Polyzogopoulou E, Karavidas I-N, et al. Safety and efficacy of salt substitution with a low sodium-potassium enriched dietary salt in patients with heart failure with reduced ejection fraction: A pilot study. Clin Nutr ESPEN. 2020;35:90–4. 31987127 10.1016/j.clnesp.2019.11.004

[54] Yin X, Paige E, Tian M, Li Q, Huang L, Yu J, et al. The proportion of dietary salt replaced with potassium-enriched salt in the SSaSS: implications for scale-up. Hypertension. 2023;80:956–65. 36628969 10.1161/HYPERTENSIONAHA.122.20115

[55] Gritter M, Wouda RD, Yeung SM, Wieërs ML, Geurts F, De Ridder MA, et al. Effects of short-term potassium chloride supplementation in patients with CKD. J Am Soc Nephrol. 2022;33:1779–89. 35609996 10.1681/ASN.2022020147 PMC9529195

[56] Morrison R, Stanford J, Lambert K. Dietary modelling to explore the impact of potassium chloride replacement for sodium in bread for adults with chronic kidney disease. Nutrients. 2021;13:2472. 34371980 10.3390/nu13072472 PMC8308590

[57] Ajenikoko A, Ide N, Shivashankar R, Ge Z, Marklund M, Anderson C, et al. Core strategies to increase the uptake and use of potassium-enriched low-sodium salt. Nutrients. 2021;13:3203. 34579080 10.3390/nu13093203 PMC8466693

[58] Yin X, Tian M, Sun L, Webster J, Trieu K, Huffman MD, et al. Barriers and facilitators to implementing reduced-sodium salts as a population-level intervention: A qualitative study. Nutrients. 2021;13:3225. 34579109 10.3390/nu13093225 PMC8471368

[59] Bhat S, Marklund M, Henry ME, Appel LJ, Croft KD, Neal B, et al. A systematic review of the sources of dietary salt around the world. Adv Nutr. 2020;11:677–86. 31904809 10.1093/advances/nmz134 PMC7231587

[60] World Health Organization. WHO STEPS Instrument Question-by-Question Guide (Core and Expanded). 2020. p. 1–18. https://www.who.int/teams/noncommunicable-diseases/surveillance/systems-tools/steps/manuals.

