# Supplementary material for: Knowledge, attitudes, and behaviours related to reduced-sodium salt: a systematic review
Source: J Hum Hypertens. 2025 Nov 27;40(1):1–9. doi: 10.1038/s41371-025-01098-2 (PMC12807862; doi:10.1038/s41371-025-01098-2)
Supplement: Supplementary file 1 — Supplementary Material [file 41371_2025_1098_MOESM1_ESM.docx]

**Supplementary Information**

**Supplementary Table 1.** Preferred Reporting Items for Systematic Reviews and Meta-Analyses (PRISMA) 2020 checklist^a^

| **Checklist item number** | **Section/topic** | **Checklist item** | **Reported on page number** |
| --- | --- | --- | --- |
| **Title** | | | |
| 1 | Title | Identify the report as a systematic review. | 1 |
| **Abstract** | | | |
| 2 | Abstract | See the PRISMA 2020 for Abstracts checklist.  Title/Title: Identify the report as a systematic review.  Background/Objectives: Provide an explicit statement of the main objective(s) or question(s) the review addresses  Methods/Eligibility criteria: Specify the inclusion and exclusion criteria for the review.  Methods/Information sources: Specify the information sources (e.g. databases, registers) used to identify studies and the date when each was last searched.  Methods/Risk of bias: Specify the methods used to assess risk of bias in the included studies.  Methods/Synthesis of results: Specify the methods used to present and synthesize results.  Results/Included studies: Give the total number of included studies and participants and summarize relevant characteristics of studies.  Results/ Synthesis of results: Present results for main outcomes, preferably indicating the number of included studies and participants for each. If meta-analysis was done, report the summary estimate and confidence/credible interval. If comparing groups, indicate the direction of the effect (i.e. which group is favored).  Discussion/Limitation of evidence: Provide a brief summary of the limitations of the evidence included in the review (e.g. study risk of bias, inconsistency and imprecision).  Discussion/Interpretation: Provide a general interpretation of the results and important implications  Other/Funding: Specify the primary source of funding for the review.  Other/Registration: Provide the register name and registration number. | 1 |
| **Introduction** | | | |
| 3 | Rationale | Describe the rationale for the review in the context of existing knowledge. | 1 |
| 4 | Objective | Provide an explicit statement of the objective(s) or question(s) the review addresses. | 1 |
| **Methods** | | | |
| 5 | Eligibility criteria | Specify the inclusion and exclusion criteria for the review and how studies were grouped for the syntheses | 2 |
| 6 | Information sources | Specify all databases, registers, websites, organizations, reference lists and other sources searched or consulted to identify studies. Specify the date when each source was last searched or consulted. | 2 |
| 7 | Search strategy | Present the full search strategies for all databases, registers and websites, including any filters and limits used. | 2, Supplementary Table 2 |
| 8 | Selection process | Specify the methods used to decide whether a study met the inclusion criteria of the review, including how many reviewers screened each record and each report retrieved, whether they worked independently, and if applicable, details of automation tools used in the process. | 2 |
| 9 | Data collection process | Specify the methods used to collect data from reports, including how many reviewers collected data from each report, whether they worked independently, any processes for obtaining or confirming data from study investigators, and if applicable, details of automation tools used in the process | 2-3 |
| 10a | Data items | List and define all outcomes for which data were sought. Specify whether all results that were compatible with each outcome domain in each study were sought (e.g. for all measures, time points, analyses), and if not, the methods used to decide which results to collect. | 2 |
| 10b |  | List and define all other variables for which data were sought (e.g. participant and intervention characteristics, funding sources). Describe any assumptions made about any missing or unclear information. | 2-3 |
| 11 | Study risk of bias assessment | Specify the methods used to assess risk of bias in the included studies, including details of the tool(s) used, how many reviewers assessed each study and whether they worked independently, and if applicable, details of automation tools used in the process. | 3, Supplementary Material 1 |
| 12 | Effect measures | Specify for each outcome the effect measure(s) (e.g. risk ratio, mean difference) used in the synthesis or presentation of results | N/A |
| 13a | Synthesis methods | Describe the processes used to decide which studies were eligible for each synthesis (e.g. tabulating the study intervention characteristics and comparing against the planned groups for each synthesis (item #5)). | N/A |
| 13b |  | Describe any methods required to prepare the data for presentation or synthesis, such as handling of missing summary statistics, or data conversions. | N/A |
| 13c |  | Describe any methods used to tabulate or visually display results of individual studies and syntheses. | N/A |
| 13d |  | Describe any methods used to synthesize results and provide a rationale for the choice(s). If meta-analysis was performed, describe the model(s), method(s) to identify the presence and extent of statistical heterogeneity, and software package(s) used. | N/A |
| 13e |  | Describe any methods used to explore possible causes of heterogeneity among study results (e.g. subgroup analysis, meta-regression) | N/A |
| 13f |  | Describe any sensitivity analyses conducted to assess robustness of the synthesized results. | N/A |
| 14 | Reporting bias assessment | Describe any methods used to assess risk of bias due to missing results in a synthesis (arising from reporting biases). | N/A |
| 15 | Certainty  assessment | Describe any methods used to assess certainty (or confidence) in the body of evidence for an outcome | N/A |
| **Results** | | | |
| 16a | Study selection | Describe the results of the search and selection process, from the number of records identified in the search to the number of studies included in the review, ideally using a flow diagram. | 3 |
| 16b |  | Cite studies that might appear to meet the inclusion criteria, but which were excluded, and explain why they were excluded. | 3-4 |
| 17 | Study characteristics | Cite each included study and present its characteristics. | 4-6 Supplementary Table 3 & 4 |
| 18 | Risk of bias in studies | Present assessments of risk of bias for each included study. | 6, Supplementary Table 7-11 |
| 19 | Results of individual studies | For all outcomes, present, for each study: (a) summary statistics for each group (where appropriate) and (b) an effect estimate and its precision (e.g. confidence/credible interval), ideally using structured tables or plots. | 5-6, Supplementary Table 5 & 6 |
| 20a | Results of syntheses | For each synthesis, briefly summarize the characteristics and risk of bias among contributing studies. | N/A |
| 20b |  | Present results of all statistical syntheses conducted. If meta-analysis was done, present for each the summary estimate and its precision (e.g. confidence/ credible interval) and measures of statistical heterogeneity. If comparing groups, describe the direction of the effect. | N/A |
| 20c |  | Present results of all investigations of possible causes of heterogeneity among study results. | N/A |
| 20d |  | Present results of all sensitivity analyses conducted to assess the robustness of the synthesized results. | N/A |
| 21 | Reporting biases | Present assessments of risk of bias due to missing results (arising from reporting biases) for each synthesis assessed. | N/A |
| 22 | Certainty of evidence | Present assessments of certainty (or confidence) in the body of evidence for each outcome assessed. | N/A |
| **Discussion** | | | |
| 23a | Discussion | Provide a general interpretation of the results in the context of other evidence. | 6-7 |
| 23b |  | Discuss any limitations of the evidence included in the review. | 6-7 |
| 23c |  | Discuss any limitations of the review processes used. | 6-7 |
| 23d |  | Discuss implications of the results for practice, policy, and future research. | 7 |
| **Other information** | | | |
| 24a | Registration and protocol | Provide registration information for the review, including register name and registration number, or state that the review was not registered. | 1 |
| 24b |  | Indicate where the review protocol can be accessed, or state that a protocol was not prepared. | 1 |
| 24c |  | Describe and explain any amendments to information provided at registration or in the protocol. | N/A |
| 25 | Support | Describe sources of financial or non-financial support for the review, and the role of the funders or sponsors in the review. | 9 |
| 26 | Competing interests | Declare any competing interests of review authors. | 9 |
| 27 | Availability of data, code and other materials | Report which of the following are publicly available and where they can be found: template data collection forms; data extracted from included studies; data used for all analyses; analytic code; any other materials used in the review. | 7 |

1. Checklist from Page MJ, McKenzie JE, Bossuyt PM, Boutron I, Hoffmann TC, Mulrow CD, et al. The PRISMA 2020 statement: an updated guideline for reporting systematic reviews. BMJ 2021;372:n71. doi: 10.1136/bmj.n71

**Supplementary Table 2.** Example search strategy utilised in PubMed bibliographic database

| The following search strategy was utilised within PubMed electronic bibliographic database |
| --- |
| ("salt substitut*"[Title/Abstract] OR “salt replac*”[Title/Abstract] OR "reduced sodium salt"[Title/Abstract] OR "low sodium salt"[Title/Abstract] OR "sodium free salt"[Title/Abstract] OR "healthy salt"[Title/Abstract] OR "potassium salt"[Title/Abstract] OR "potassium enriched salt"[Title/Abstract] OR "lite salt"[Title/Abstract] OR "kcl salt"[Title/Abstract] OR "potassium chloride salt"[Title/Abstract] OR "mineral salt"[Title/Abstract]) AND ("consumer"[Title/Abstract] OR "user"[Title/Abstract] OR "public"[Title/Abstract] OR “knowledge”[Title/Abstract] OR “perception”[Title/Abstract] OR “perspective”[Title/Abstract] OR “attitude”[Title/Abstract] OR “awareness”[Title/Abstract] OR “belief”[Title/Abstract] OR “practice”[Title/Abstract] OR “behavior”[Title/Abstract] OR “behaviour”[Title/Abstract] OR “enable*”[Title/Abstract] OR “barrier”[Title/Abstract] OR “attitude to health”[MeSH Terms] OR “consumer behavior”[MeSH Terms] OR “perception”[MeSH Terms] OR “awareness”[MeSH Terms]) |

**Supplementary Material 1.** Adaptations made to the Newcastle Ottawa Scale (NOS)

Adaptations were made to the Newcastle Ottawa Scale (NOS) separately based on study design. For non-randomized intervention studies, adaptations were made based on the original NOS and adaptation by Herzog et al, while for cross-sectional studies adaptations were made to the adaptation by Herzog et al. Within both adaptations an additional ‘comparability’ rating was included where the study does not control for the most important factors, age or sex. For non-randomized intervention studies, adaptations to the original NOS included the addition of sample size criteria under the selection domain and statistical test criteria under the outcome domain, as outlined in the adaptation by Herzog et al. Briefly, a star is awarded if 1) the sample size is justified and satisfactory and 2) the statistical test used to analyze the data is clearly described and appropriate, and the measurement of the association is presented. Furthermore, the ascertainment of exposure criteria and assessment of outcome criteria were changed to reflect options listed in Herzog et al. as it was more relevant to the current review and outcomes of interest. Criteria for the selection of the non-exposed cohort was removed as it was not relevant to studies included in the current review where all participants were “exposed” to reduced-sodium salt for the relevant outcomes of interest.

**Supplementary Table 3.** Characteristics of studies evaluating a reduced-sodium salt intervention^a^

| **Article** | **Country** | **Income classification^b^** | **Setting** | **Study design** | **Study cohort** | **n** | **Participant type** | **Age, y**  **Mean (SD)** | **Sex** | **Method of reduced-sodium salt** | **Composition of reduced-sodium salt** | **Comparator** | **Outcome** | **Method to assess outcome** | **Results** |
| --- | --- | --- | --- | --- | --- | --- | --- | --- | --- | --- | --- | --- | --- | --- | --- |
| Land et al (2016) | Australia | High | Population level | Non-randomized intervention | Salt Swap Lithgow | Random followed from baseline: 101  Random new: 106  Volunteer followed from baseline: 36  Volunteer new: 329 | Consumers | Random followed from baseline: 64  Random new: 60  Volunteer followed from baseline: 60  Volunteer new: 52 | Random followed from baseline:  F: 56%;  M: 44%  Random new:  F: 61%;  M: 39%  Volunteer followed from baseline:  F: 61%;  M: 39%  Volunteer new:  F: 58%;  M: 42% | Providing free of charge at local cafes, restaurant, gov buildings and medical centers | 136mg sodium and 176mg potassium per serving (0.8 g) | N/A | Behavior: Use | Questionnaire | At follow-up, 26% of participants reported using the salt substitute that was provided |
| Wang et al (2016) | China | Upper-middle | Population level | Randomized-controlled | China Rural Health Initiative Sodium Reduction Study (CRHI-SRS) | Education 0 years: 329 | Consumers | 63.8 (9.6) | F: 75.4%;  M: 24.6% | Available for purchase in grocery stores with/ without subsidy | Not reported | N/A | Behavior: Use | Questionnaire | Intervention vs. control:  53% vs. 7%  Subsidy vs. no subsidy:  72% vs. 36% |
|  |  |  |  |  |  | Education 1-6 years: 697 |  | 59.5 (10.7) | F: 52.5%;  M: 47.5% |  |  |  |  |  | Intervention vs. control:  65% vs. 7%  Subsidy vs. no subsidy:  76% vs. 50% |
|  |  |  |  |  |  | Education 7-9 years: 704 |  | 47.7 (13.5) | F: 41.5%;  M: 58.5% |  |  |  |  |  | Intervention vs. control:  65% vs. 5%  Subsidy vs. no subsidy:  80% vs. 47% |
|  |  |  |  |  |  | Education >9 years: 171 |  | 44.8 (15.2) | F: 30.4%;  M: 69.6% |  |  |  |  |  | Intervention vs. control:  59% vs. 7%  Subsidy vs. no subsidy:  83% vs. 31%" |
| Chu et al (2021) | China | Upper-middle | Population level | Randomized-controlled (process evaluation) | China Rural Health Initiative Sodium Reduction Study (CRHI-SRS) | 45 | Consumers | Not reported | Not reported | Available for purchase in grocery stores with/ without subsidy | Not reported | N/A | Attitude: Taste | Semi-structured interview | Some participants felt the taste was less salty than regular salt |
|  |  |  |  |  |  |  |  |  |  |  |  |  | Attitude: Accessibility |  | Some participants felt it was more expensive |
|  |  |  |  |  |  |  |  |  |  |  |  |  | Behavior: Use |  | 82.2% used a low sodium salt |
| Eyles et al (2023) | New Zealand | High | Population level | Randomized-controlled | Salt Alternative Study (SALTS) | 84 | Consumers | 54 (13) | F: 45%;  M: 43%; Non-binary or not specified: 12% | Provided as is for cooking/ seasoning | 75% NaCl + 25% KCl | N/A | Attitude: Taste | Questionnaire | 4/20 (20%) of those participants that reported using less than half or none of the salt substitute stated this was because the taste was unacceptable |
| Li et al (2009) | China | Upper-middle | Population level | Randomized-controlled | China Salt Substitute Study (CSSS) | 608 | Consumers | 60.0 | F: 56%;  M: 44% | Provided as is for cooking/ seasoning | 65% NaCl + 25% KCl + 10% MgSO4 | 100% NaCl | Attitude: Taste | 100mm visual analogue scale | Overall difference between the randomized groups for saltiness (Primary - unweighted): 0.02 (p=0.09)  Overall difference between the randomized groups for saltiness (Secondary - weighted): 0.8 (p=0.80)  Overall difference between the randomized groups for flavor: (Primary - unweighted): -1.4 (p=0.08)  Overall difference between the randomized groups for flavor: (Secondary - weighted): -1.8 (p=0.045) |
|  |  |  |  |  |  |  |  |  |  |  |  |  | Attitude: Acceptability |  | Overall difference between the randomized groups for acceptability: (Primary - unweighted): -1.3 (p=0.97)  Overall difference between the randomized groups for acceptability: (Secondary - weighted): -1.6 (p=0.09) |
| Liu et al (2021) | China | Upper-middle | Population level | Randomized-controlled (process evaluation) | Salt Substitute and Stroke Study (SSaSS) | 30 | Consumers | 70.3 (6.0) | F: 60%;  M: 40% | Provided as is for cooking/ seasoning | 75% NaCl + 25% KCl | N/A | Attitude: Taste | Semi-structured interview | Many of the interviewees suggest the taste was highly acceptable |
|  |  |  |  |  |  |  |  |  |  |  |  |  | Attitude: Accessibility |  | Participants identified low availability and available salt substitute not readily accessible; salt substitute cannot be found in village grocery stores.  Participants were sensitive to higher prices of salt substitute; prefer the regular salt owing to lower price than the salt substitute |
|  |  |  |  |  |  |  |  |  |  |  |  |  | Behavior: Willingness to use |  | Many of the interviewees expressed a willingness to consume the salt substitute |
| Yu et al (2021) | India | Lower-middle | Population level | Randomized-controlled | Salt Substitute in India Study (SSiIS) | 502 | Consumers | 61.6 (12) | F: 58.8%;  M: 41.2% | Provided as is for cooking/ seasoning | 70% NaCl + 30% KCl | 100% NaCl | Attitude: Taste | Questionnaire | Participants reported that they enjoyed the taste of the study salt; on a scale of 1-10, the mean and SD for the salt substitute group were 8.6 (1.2) and 8.5 (1.1) for the regular salt group |
| Barros et al (2015) | Brazil | Upper-middle | Community level | Randomized-controlled | - | 35 | Consumers | 55.5 (7.4) | F: 65.7%;  M: 34.3% | Provided as is for cooking/ seasoning | Light salt: 130mg of sodium + 346mg of potassium per gram | Regular salt: 390mg of sodium per gram | Attitude: Acceptability | Unknown Self-report | Light salt had low acceptance by 89.5% of intervention participants |
|  |  |  |  |  |  |  |  |  |  |  |  |  | Attitude: Taste |  | Low acceptance of light salt was due to its peculiar taste, where participants claimed there were taste changes in the prepared foods |
| Charlton et al (2008) | South Africa | Upper-middle | Community level | Randomized-controlled | - | IG: 40  CG: 40 | Consumers | IG: 61.8 (6.6)  CG: 60.4 (7.4) | IG:  F: 82.5%;  M: 17.5%  CG:  F: 85.0%;  M: 15.0% | Provided as is for cooking/ seasoning | Solo salt: 41% NaCl + 41% KCl + 17% Mg salt | 100% NaCl | Attitude: Taste | 5-point likert scale | The salt replacement was reported to taste less acceptable than the normal products among 75% of intervention participants |
| Pan et al (2017) | Taiwan | High | Community level | Randomized-controlled | - | Na salt: 99 K salt: 97 K/Mg salt: 96 | Consumers | Na salt: 64.8 (10.3)  K salt: 64.4 (9.8)  K/Mg salt: 64.7 (9.9) | Na salt:  F: 32.3%  M: 67.7%  K salt:  F: 42.3%  M: 57.7%  K/Mg salt:  F:34.7%  M: 65.3% | Provided as is for cooking/ seasoning | K salt: 50% NaCl + 50% KCl  K/Mg salt: 42.85% NaCl + 42.85% KCl + 14.3% MgSO4 | Na salt; 100% NaCl | Attitude: Taste | Unknown Self-report | The K/Mg salt was least liked by the study participants (16.2% dislike) compared with K salt (13.3% dislike) and Na salt (11.5% dislike) because of its slightly bitter taste |
| Pietinen et al (1981) | Finland | High | Community level | Randomized-controlled | - | IG: 27 families  CG: 31 families | Consumers | IG mother: 38.9  IG father: 42.5  CG mother: 42.0  CG father: 43.8  All children were 13 years of age | Parents (both IG and CG):  F: 50%;  M: 50%  Children:  IG:  F: 41.9%;  M: 58.1%  CG:  F: 34.6%;  M: 65.4% | Provided as is for cooking/ seasoning | 65% NaCl + 25% KCl + 10% MgSO4 | 100% NaCl | Attitude: Taste | Questionnaire | 48% of CG mothers and 36% of IG mothers stated that food tasted as salty as normal    64% of the IG mothers considered food less salty and 0% saltier than normal  19% of IG mothers reported a bitter or somehow strange taste |
| Crouch et al (2023) | South Africa | Upper-middle | Community level | Non-randomized intervention | - | 56 | Consumers | Median: 21 | F: 54.4%;  M: 45.6% | Provided and tested as is with crackers | Cerebos 1: 65% NaCl + 35% KCl  Cerebos 2: 50% NaCl + 50% KCl  Lo Salt: 34% NaCl + 66% KCl  Nature’s Source: 100% KCl | 100% NaCl | Attitude: Taste | Questionnaire | Taste Ranking:  100% NaCl had the highest top-2-box score for taste ranking (54.9 %) and taste perception (69.6%).  Top-2-box score proportions were significantly different between 100% NaCl and 35% KCl (p=0.012), 66% KCl (p≤0.001), and 100% KCl (p≤0.001). There was no difference with 50% KCl. Bottom-2-box score proportions were significantly different between 100% NaCl and 100% KCl (p≤0.001).  45% of participants ranked 50% KCl as either “fantastic” or “really good”. 12% of participants ranked the same for 100% KCl. 51% of participants ranked 100% KCl as “not very good” or “awful”. 22% of participants ranked the same for 50% KCl. Responses were similar on either side for 35% KCl and 66% KCl.  Taste perception:  62% of participants indicated they liked and would be happy to use the 50% KCl or felt it tasted like normal seasoning. 71 % of participants indicated they would eat but did not like or would not eat the 100% KCl. Responses for 35% KCl and 66% KCl were split equally.  Both top- and bottom-2-box score proportions were significantly different between 100% NaCl and 35% KCl (p=0.034 both), 66% KCl (p=0.034 both), and 100% KCl (p≤0.001 both). There was no difference with 50% KCl. |
|  |  |  |  |  |  |  |  |  |  |  |  |  | Behavior: Willingness to use |  | 57% ranked 50% KCl the most or second most likely to use while 56 % of participants ranked the 100% KCl and 54% ranked the 66% KCl as the least or second least likely to use.  Both top- and bottom-2-box score proportions were significantly different between 100% NaCl and 66% KCl (p≤0.001 both), and 100% KCl (p≤0.001 both). There was no difference with 35% KCl or 50% KCl. |
| Sinopoli & Lawless (2012) | United States | High | Community level | Non-randomized intervention | - | 21 | Consumers | Not reported | F: ~50%;  M: ~50% | Provided and tested as is in water solutions | 100% KCl | 100% NaCl | Attitude: Taste | Focus groups | The top 10 words selected to describe the KCl solution in descending order were bitter (39%), chemical, metallic, plastic, salty, astringent, drying, sour, moldy, and no taste (11%). |
|  |  |  |  |  |  | 100 | Consumers | <20 to >60 years | F: 50%;  M: 50% | Provided and tested as is in water solutions | 100% KCl | 100% NaCl | Attitude: Taste | Questionnaire | Less participants selected salty to describe the KCl solution (17%) compared to the control (92%).  More participants selected bitter to describe the KCl solution (39%) compared to the control (7%).  More participants selected chemical to describe the KCl solution (31%) compared to the control (16%).  More participants selected metallic to describe the KCl solution (28%) compared to the control (8%). |
| Lazo-Porras et al (2023) | Peru | Upper-middle | Community level | Non-randomized intervention | SALT project | 60 | Consumers | Median (IQR)  With HTN: 66 (53-70)  Without HTN: 35 (29-50) | F: 100% | Provided as is for cooking/ seasoning and also provided to owners of small shops, bakeries, local kitchens, street vendors and restaurant | Salt Liz: 75% NaCl + 25% KCl | N/A | Attitude: Acceptability | Interview | Acceptability of the Salt Liz was high among participants.  Learning about Salt Liz’s effects on health influenced acceptability among participants  Beyond health, participants in the four villages accepted the Salt Liz because it was a higher quality salt. |
|  |  |  |  |  |  |  |  |  |  |  |  |  | Attitude: Taste |  | Most participants reported that Salt Liz was not as salty as regular salt and noticed a change in the flavor of foods, however it took about 2 weeks to adapt to the changed flavor and saltiness.  Participants who were already accustomed to foods low in salt and seasoning did not notice differences in taste.  Several participants reported going through an adjustment period including becoming accustomed to foods with lower sodium content and adjusting the amount of salt added to give the desired flavor. |
|  |  |  |  |  |  |  |  |  |  |  |  |  | Behavior: Willingness to use |  | 56/60 (93%) participants said they would like to buy the salt substitute if it becomes available for purchase |
| Lloyd-Sherlock et al (2018) | South Africa | Upper-middle | Community level | Non-randomized intervention | - | 13 | Consumers | >60 years | F: 46.2%;  M: 53.8% | Provided as is for cooking/ seasoning | Not reported | N/A | Behavior: Willingness to use | Focus groups | All participants were very keen to continue using the product if it could be made available |
|  |  |  |  |  |  |  |  |  |  |  |  |  | Attitude: Acceptability |  | Overall, participants had high acceptability of the low sodium salt product |
| Hueston (1989) | United States | High | Community level | Non-randomized intervention | - | 10 | Consumers | 51.6 (8.5) | F: 50%;  M: 50% | Provided as is for seasoning or addition to fruit juice | No Salt, Norcliff-Thayer: 100% KCl | Potassium supplement | Attitude: Taste | Questionnaire | 8/9 subjects rated the taste of the salt substitute as worse or much worse than their usual potassium supplement |
|  |  |  |  |  |  |  |  |  |  |  |  |  | Attitude: Preference |  | Only one subject preferred the salt substitute, even when informed that cost is 1/10^th^ the cost of the supplements |
| Maleki et al (2016) | Iran | Lower-middle | Community level | Randomized-controlled | - | 100 | Consumers | 38.1 (10.7) | F: 55%;  M: 45% | Provided and tested as is | 6 different salts:  95% NaCl + 5% KCl;  90% NaCl + 10% KCl;  85% NaCl + 15% KCl;  80% NaCl +  20% KCl;  75% NaCl + 25% KCl;  70% NaCl + 30% KCl | 100% NaCl | Attitude: Acceptability | Questionnaire | More than 80 % of participants either did not distinguish between the two salts provided even in high potassium-enriched salts or preferred potassium-enriched salt (p<0.001) |
|  |  |  |  |  |  |  |  |  |  |  |  |  | Attitude: Preference |  | The number of participants who preferred potassium-enriched salt was greater than the number of participants who preferred regular sodium chloride salt in all concentrations but 10% KCl (p=0.001 5-20% KCl; p=0.04 25% KCl and p=0.014 30% KCl) |
| Sopko et al (1977) | United States | High | Community level | Non-randomized intervention | - | 28 | Consumers | Not reported | Not reported | Provided and tested as is | Adolph's Salt: 12mEq/g K + 0.18mg/g Ca  Co-Salt: 12.2mEq/g K + 0.04mg/g Ca  Diasal: 10.7mEq/g K + 0.54mg/g Ca + 0.07mEq/g Mg  Feather weight K salt: 11.2mEq/g K + 1.14mg/g Ca + 0.3mg/g Ph  Neocurtasal: 12mEq/g K + 5.95mg/g Ca + 1.6mg/g Ph  Sweet and Low: 12.4mEq/g K + 0.54mg/g Ca  Morton Salt: 13mEq/g K + 3.48mg/g Ca + 1.9mg/g Ph  Feather weight seasoned: 10.3mEq/g K + 2.94mg/g Ca + 0.14mEq/g Mg + 1.2mg/g Ph | N/A | Attitude: Taste | Questionnaire | For salty taste, Neocurtasal and Featherweight K Salt Substitute were statistically different than all other products, favors salty taste (p=0.1). The most salty was Neocurtasal, the least salty was featherweight seasoned  For bitter taste, Neocurtasal, Featherweight seasoned Salt Substitute and Morton Salt Substitute were statistically different than all other products, favors bitter taste (p=0.05). The most bitter was Neocurtasal, the least bitter was Co-Salt |
|  |  |  |  |  |  |  |  |  |  |  |  |  | Attitude: Preference |  | For overall preference, Neocurtasal, Morton Salt Substitute and Featherweight “K” Salt Substitute were statistically different than all other products, favors most preferred (p=0.01). The most preferred was Neocurtasal, the least preferred was Adolph’s Salt Substitute |

1. CG: control group; F: female; HTN: hypertension; IG: intervention group; KAB: knowledge, attitude, behaviors; M: male
2. Income classification by country according to The World Bank 2023

**Supplementary Table 4.** Characteristics of descriptive studies without the provision of reduced-sodium salt^a^

| **Article** | **Country** | **Income classification^b^** | **Setting** | **Study design** | **Study cohort** | **n** | **Participant type** | **Age, y**  **Mean (SD)** | **Sex** | **Outcome** | **Method to assess outcome** | **Results** |
| --- | --- | --- | --- | --- | --- | --- | --- | --- | --- | --- | --- | --- |
| Du et al (2022) | China | Upper-middle | Population level | Cross-sectional | Salt Reduction and Hypertension Prevention  Project (SRHPP) | 7512 | Consumers | 44.8 (14) | F: 50.1%; M: 49.9% | Knowledge: Awareness of reduced-sodium salt | Questionnaire | 30.0% know about low-sodium salt.  Significant difference between males and females in urban areas; greater knowledge among females (p<0.001).  Significant difference between urban and rural areas; greater knowledge among urban areas (p<0.001). |
|  |  |  |  |  |  |  |  |  |  | Knowledge: Awareness of health effects |  | 20.8% know that low-sodium salt helps control BP.  Significant difference between males and females; greater knowledge among females (p<0.001 urban, p<0.05 rural).  Significant difference between urban and rural areas; greater knowledge among urban areas (p<0.001). |
|  |  |  |  |  |  |  |  |  |  | Behavior: Use |  | 15.7% are using or used low-sodium salt.  Significant difference between males and females in urban areas; greater use among females (p<0.001).  Significant difference between urban and rural areas; greater use among urban areas (p<0.001). |
| Han et al (2022) | China | Upper-middle | Population level | Cross-sectional | - | 7665 | Consumers | 54.6 (13.3) | F: 57.4%; M: 42.6% | Behavior: Use | Questionnaire | 10.37% of participants use  low-sodium salt |
| Yang et al (2021) | China | Upper-middle | Population level | Cross-sectional | China Healthy Lifestyle for All Campaign (CHLA) 2019 | 2109 | Consumers | ≤45 years:  65.3% | F: 70.2%; M: 29.8% | Behavior: Use | Questionnaire | 35.8% regularly used low-sodium salt |
|  |  |  |  |  | China Healthy Lifestyle for All Campaign (CHLA) 2020 | 12,732 |  | ≤45 years:  60.6% | F: 70.8%; M: 29.2% |  |  | 43.4% regularly used low-sodium salt |
| Zhang, P et al (2023) | China | Upper-middle | Population level | Cross-sectional | Action on Salt China (ASC) consisting of 3 different studies (CIS, HIS and AIS) | 4,000 (CIS: 2,642; HIS: 766; AIS: 592) | Consumers | 49.0 (12.8) | F: 57.3%; M: 42.7% | Knowledge: Awareness of reduced-sodium salt | Questionnaire | 32% of participants were aware of or reported having ever heard of LSSS.  Participants living in cities had higher awareness of LSSS than participants living in rural areas (p<0.001).  The awareness of LSSS was highest among participants in the 18–44 age group, compared to the 45–60 and over 60 age groups (p<0.001).  The higher the education level, the higher the awareness of LSSS (p<0.001).  Participants with normal blood pressure had a higher awareness of LSSS than those with hypertension (p<0.001).  There was no difference in the awareness of LSSS between males and females. |
|  |  |  |  |  |  |  |  |  |  | Behavior: Use |  | 11.7% of participants were aware of and using LSSS, while 20.3% were aware of but not using LSSS.  Participants living in cities had a higher use rate of LSSS than participants living in rural areas (p<0.001).  The use of LSSS was the highest among participants in the 18–44 age group, compared to the 45–60 and over 60 age groups (p<0.001).  The higher the education level, the higher the use of LSSS (p<0.001).  Participants with normal blood pressure had a higher use of LSSS than those with hypertension (p<0.001).  There was no difference in the use of LSSS between males and females. |
| Zhang, W et al (2023) | China | Upper-middle | Population level | Cross-sectional | China Chronic Disease and Nutrition Surveillance (CCDNS) | 179,834 | Consumers | 43.9 (14.4) | F: 51.7%; M: 48.3% | Behavior: Use | Questionnaire | 12.2% of participants used LSSS (16.0% in established hypertension group, 12.3% in no hypertension group, and 10.1% in newly diagnosed hypertension group.  More females used LSSS than males (p<0.0001).  Participants in rural areas had lower LSSS use in all hypertension groups compared to urban areas (p<0.0001)  LSSS use increased with increasing education level (p<0.0001).  LSSS use was lowest among participants ≥60 years across all hypertension groups (p<0.01).  LSSS use was higher among those living in the South than those in the North in established hypertension group (p<0.05).  LSSS use was higher in provinces where salt reduction campaigns had been implemented before 2015 (p=0.0002). |
| Yu et al (2021) | India | Lower-middle | Population level | Randomized-controlled | Salt Substitute in India Study (SSiIS) | 502 | Consumers | 61.6 (12) | F: 58.8%;  M: 41.2% | Knowledge: Awareness of reduced-sodium salt | Questionnaire | None of the participants had heard about reduced-sodium salt at baseline |
| Sehgal et al (2023) | India | Lower-middle | Community level | Cross-sectional | Promoting Uptake of Low SodiUm Iodized Salt by Rural And Urban HousehoLds in India study (PLURAL) | Consumers: 20 (interviews) and 40 (across 4 focus groups); Retailers: 26 (interviews) and 20 (across 2 focus groups); Influencers: 16 (interviews) | Consumers, retailers, and influencers (public healthcare staff (doctors, auxiliary nurse midwives, accredited social health activists), members of women’s self-help groups, village heads and members of resident welfare associations) | 47 | F: 38%; M: 62% | Knowledge: Awareness of reduced-sodium salt | Semi-structured interview and focus groups | Consumers: Most of the participants were unaware of LSIS. Consumers became aware of LSIS for the first time during their participation in this study  Influencers: Lack of awareness of LSIS is a major barrier to its use. Most stakeholders, which included healthcare providers, were unaware of LSIS. |
|  |  |  |  |  |  |  |  |  |  | Attitude: Accessibility |  | Consumers: Cost was reported as a major barrier to purchase LSIS, but subsidy as an intervention to reduce the price of LSIS was welcomed. The cost of LSIS was a significant factor in the purchase decision of consumers. The cost of LSIS was comparatively higher compared to regular salt. Participants agreed that a subsidy would be important to offset the extra cost of LSIS  Influencers: Lack of availability of LSIS with high cost is a major barrier to its use |
|  |  |  |  |  |  |  |  |  |  | Attitude – Recommendation of reduced-sodium salt |  | Consumers: Healthcare professionals were mentioned as the most trusted source for promoting LSIS  Influencers: A need for interpersonal communication to convey the message regarding LSIS was highlighted. In the case of rural areas, it is important to involve community leaders at the village level. |
|  |  |  |  |  |  |  |  |  |  | Attitude - Taste |  | Consumers: According to many participants, taste was the main factor which hindered the transitioning from regular salt to a healthier salt alternative |
| Fathima et al (2018) | India | Lower-middle | Community level | Cross-sectional | - | 165 | HCP: Doctors | 34.6 (10.7) | F: 40%;  M: 60% | Knowledge: Awareness of reduced-sodium salt | Semi-structured interview | 70.9% of participants were aware about the availability of various brands of LSSS, but 87.2% could not name any of the LSSS available in the Indian market  For access to LSSS, 85.5% said that it is available in retail shops, 64.2% reported its availability in medical shops, and 73.3% of participants said that it can be purchased from online markets  Only 17.6% of participants knew that potassium is used for reduction in sodium content in these salts |
|  |  |  |  |  |  |  |  |  |  | Knowledge: Awareness of use in specific disease states |  | 43.6% did not know exact indication for the use of LSSS, 52.8% reported that it is used in hypertension. 2.4% and 1.2% identified chronic kidney disease and coronary artery disease as indication for use, respectively.  71.5% did not know diseases where LSSS were contraindicated. <10% identified SIADH/orthostatic hypotension, hyponatremia, hypertension, hypokalemia, cardiovascular disease, and renal disease as diseases where LSSS is contraindicated.  Participants identified the use of LSSS safe in patients with pedal edema (39.4%), renal failure (44.8%), diabetes (47.3%), potassium sparing diuretics (24.8%), ACE inhibitors (22.4%), frequent NSAID users (21.8%), and obstructive uropathy (21.8%) |
|  |  |  |  |  |  |  |  |  |  | Behavior: Prescription |  | Only 18.8% had prescribed them during their clinical practice |
| Albert et al (2002) | United States | High | Community level | Cross-sectional | - | 300 | HCP: Nurses | Not reported | Not reported | Knowledge: Awareness of use in specific disease states | 20-item questionnaire | 52.3% correctly answered "False" to the following  statement: It is ok to use potassium-based salt substitutes (like “No-Salt” or “Salt Sense”) to season food  15.0% of participants requested more information regarding tis question |
| Washburn et al (2005) | United States | High | Community level | Cross-sectional | - | 51 | HCP: Nurses | Not reported | Not reported | Knowledge: Awareness of use in specific disease states | 20-item questionnaire | 41.2% correctly answered “False” to the following statement: It is ok to use potassium-based salt substitutes (like “NoSalt” or “Salt Sense”) to season food  17.6% of participants requested more information regarding this question |
| Fowler (2012) | United States | High | Community level | Non-randomized intervention | - | 15 | HCP: Nurses | Not reported | Not reported | Knowledge: Awareness of use in specific disease states | Questionnaire | At baseline, 66% of participants correctly answered "False" to the following statement:  It is okay to use potassium-based salt substitutes (e.g., “no-salt” or “salt-sense”) to season food |
| Yehle et al (2012) | United States | High | Community level | Non-randomized intervention | - | 235 | HCP:  Nurse students | Majority were 20 years old, not otherwise specified | Majority were female, not otherwise specified | Knowledge: Awareness of use in specific disease states | Questionnaire | At baseline, 44.9% of participants correctly answered "False" to the following statement: It is acceptable to take potassium-based salt substitute |

1. AIS: App-based salt reduction program for primary school children and their families; CIS: Community-based comprehensive salt reduction intervention study; F: female; HCP: healthcare professional; HIS: Home-cook salt reduction intervention study; HTN: hypertension; M: male
2. Income classification by country according to The World Bank 2023

**Supplementary Table 5.** Perceptions of overall acceptability following a reduced-sodium salt intervention

| **Study (year), country (ref)** | **n participants** | **Length of follow-up** | **Salt composition tested** | **Participant blinding (yes/no)** | **Overall acceptability outcome** | **Overall** |
| --- | --- | --- | --- | --- | --- | --- |
| Li et al (2009), China | 608 | 12-months | 65% NaCl + 25% KCl + 10% MgSO4 | Yes | No differences in overall acceptability of home-cooked meals using reduced-sodium salt compared to regular salt | Positive |
| Lazo-Porras et al (2023), Peru | 60 | 2-months to 23-months | 75% NaCl + 25% KCl | No | Acceptability was high among participants. | Positive |
| Lloyd-Sherlock et al (2018), South Africa | 13 | 3-months | Not reported | No | Overall, participants had high acceptability of the reduced-sodium salt | Positive |
| Maleki et al (2016), Iran | 100 | 0-weeks | 70-95% NaCl +  5-30% KCl | Yes | More than 80% of participants did not distinguish between the two salts provided even when comparing reduced-sodium salts high in potassium chloride or comparing reduced-sodium salts selected as preferred in other comparisons (p<0.001) | Positive |
| Barros et al (2015), Brazil | 35 | 4-weeks | 130mg of sodium + 346mg of potassium per gram | Yes | The reduced-sodium salt had low acceptance by 89.5% of intervention participants | Negative |

*KCl: potassium chloride; MgSO4: magnesium sulphate; NaCl: sodium chloride

**Supplementary Table 6.** Perceptions of taste following a reduced-sodium salt intervention

| **Study (year), country** | **n participants** | **Length of follow-up** | **Reduced-sodium salt composition*** | **Participant blinding (yes/no)** | **Taste outcome** | **Overall** |
| --- | --- | --- | --- | --- | --- | --- |
| Eyles et al (2023), New Zealand | 84 | 12-weeks | 75% NaCl + 25% KCl | No | 69% (44/64) of intervention participants who provided data reported using the reduced-sodium salt for half to all salt use.  Only 4/20 participants that reported using less than half or none of the reduced-sodium salt stated this was because the taste was unacceptable | Positive |
| Li et al (2009),  China | 608 | 12-months | 65% NaCl + 25% KCl + 10% MgSO4 | Yes | No differences in saltiness or flavour of home-cooked meals using reduced-sodium salt compared to regular salt | Positive |
| Liu et al (2021), China | 30 | 3-years | 75% NaCl + 25% KCl | No | The majority of interviewees suggest the taste of the reduced-sodium salt was highly acceptable | Positive |
| Yu et al (2021), India | 502 | 3-months | 70% NaCl + 30% KCl | Yes | Participants reported that they enjoyed the taste of the reduced-sodium salt. There were no taste differences to regular salt | Positive |
| Chu et al (2021), China | 45 | 18-months | Not reported | No | Some participants felt the taste of the reduced-sodium salt was less salty than regular salt | Negative |
| Barros et al (2015), Brazil | 35 | 4-weeks | 130mg of sodium + 346mg of potassium per gram | Yes | Low acceptance of the reduced-sodium salt was due to its peculiar taste, where participants claimed there were taste changes in the prepared foods | Negative |
| Sinopoli & Lawless (2012), United States | 100 | 0-weeks | 100% KCl | Yes | Less participants selected salty and more participants selected bitter, chemical and metallic to describe the reduced-sodium salt solution compared to the regular salt solution. | Negative |
| Charlton et al (2008), South Africa | 40 | 8-weeks | Solo salt: 41% NaCl + 41% KCl + 17% Mg salt | Yes | 75% of consumers reported a less than acceptable taste of the reduced-sodium salt compared to regular salt | Negative |
| Lazo-Porras et al (2023), Peru | 60 | 2-months to 23-months | Salt Liz: 75% NaCl + 25% KCl | No | Most participants reported that the reduced-sodium salt was not as salty as regular salt and noticed a change in the flavour of foods | Negative |
| Pan et al (2017), Taiwan | K salt: 97  K/Mg salt: 96  Na salt: 99 | 6-months | K salt: 50% NaCl + 50% KCl  K/Mg salt: 42.85% NaCl + 42.85% KCl + 14.3% MgSO4 | Yes | The K/Mg reduced-sodium salt was least liked by the study participants (16.2% dislike) compared with the K reduced-sodium salt (13.3% dislike) and regular salt (11.5% dislike) because of its slightly bitter taste | Negative |
| Pietinen et al (1981), Finland | 27 | 2-months | 65% NaCl + 25% KCl + 10% MgSO4 | Yes | 36% of intervention group mothers stated that food tasted as salty as normal  64% of the intervention group mothers considered the food less salty and 0% saltier than normal  19% of intervention group mothers reported a bitter or somehow strange taste | Negative |
| Hueston (1989), United States | 10 | 6-weeks | No Salt, Norcliff-Thayer: 100% KCl | Yes | 8/9 subjects rated the taste of the reduced-sodium salt as worse or much worse than their usual potassium supplement | Negative |
| Crouch et al (2023), South Africa | 56 | 0-weeks | Cerebos 1: 65% NaCl + 35% KCl  Cerebos 2: 50% NaCl + 50% KCl  Lo Salt: 34% NaCl + 66% KCl  Nature’s Source: 100% KCl | Yes | There were significant differences in taste ranking and taste perception between regular salt and Cerebos 1, Lo Salt and Nature’s Source (p<0.05), favours regular salt. There was no difference with Cerebos 2.  45% of participants ranked either “fantastic” or “really good” for Cerebos 2. 51% and 22% of participants ranked “not very good” or “awful” for Nature’s Source and Cerebos 2 respectively. Responses were mixed for Cerebos 1 and Lo Salt.  62% of participants liked and would be happy to use Cerebos 2 or felt it tasted like normal seasoning. 71% of participants would eat but did not like or would not eat Nature’s Source. Responses were split equally for Cerebos 1 and Lo Salt. | Mixed |
| Sopko et al (1977), United States | 28 | 0-weeks | Adolph's Salt: 12mEq/g K + 0.18mg/g Ca  Co-Salt: 12.2mEq/g K + 0.04mg/g Ca  Diasal: 10.7mEq/g K + 0.54mg/g Ca + 0.07mEq/g Mg  Feather weight K salt: 11.2mEq/g K + 1.14mg/g Ca + 0.3mg/g Ph  Neocurtasal: 12mEq/g K + 5.95mg/g Ca + 1.6mg/g Ph  Sweet and Low: 12.4mEq/g K + 0.54mg/g Ca  Morton Salt: 13mEq/g K + 3.48mg/g Ca + 1.9mg/g Ph  Feather weight seasoned: 10.3mEq/g K + 2.94mg/g Ca + 0.14mEq/g Mg + 1.2mg/g Ph | Yes | For salty taste, Neocurtasal and Featherweight K Salt Substitute were statistically different than all other products, favours salty taste (p=0.1). The most salty was Neocurtasal, the least salty was featherweight seasoned  For bitter taste, Neocurtasal, Featherweight seasoned Salt Substitute and Morton Salt Substitute were statistically different than all other products, favours bitter taste (p=0.05). The most bitter was Neocurtasal, the least bitter was Co-Salt | Mixed |

*Ca: calcium; K: potassium; KCl: potassium chloride; Mg: magnesium; MgSO4: magnesium sulphate; NaCl: sodium chloride; Ph: phosphate

**Supplementary Table 7.** Quality assessment of cross-sectional descriptive studies using a modified Newcastle-Ottawa Scale (NOS) tool

|  | **Selection** | | | | **Comparability** | **Outcome** | |  |
| --- | --- | --- | --- | --- | --- | --- | --- | --- |
| **Article** | **1** | **2** | **3** | **4** | **1** | **1** | **2** | **Total stars^a^** |
| Han et al (2022) | 1a) ★ | 2b) | 3a) ★ | 4b) ★ | 1c) | 1c) ★ | 2b) | 4 |
| Du et al (2022) | 1a) ★ | 2a) ★ | 3c) | 4b) ★ | 1b) ★ | 1c) ★ | 2a) ★ | 6 |
| Sehgal et al (2023) | 1c) | 2a) ★ | 3a) ★ | 4b) ★ | 1c) | 1c) ★ | 2b) | 4 |
| Yang et al (2021) | 1b) ★ | 2b) | 3c) | 4b) ★ | 1c) | 1c) ★ | 2b) | 3 |
| Zhang, P et al (2023) | 1a) ★ | 2b) | 3a) ★ | 4b) ★ | 1a) ★1b) ★ | 1c) ★ | 2a) ★ | 7 |
| Zhang, W et al (2023) | 1a) ★ | 2a) ★ | 3a) ★ | 4b) ★ | 1a) ★1b) ★ | 1c) ★ | 2a) ★ | 8 |
| Albert et al (2002) | 1b) ★ | 2b) | 3c) | 4a) ★★ | 1c) | 1c) ★ | 2b) | 4 |
| Washburn et al (2005) | 1c) | 2b) | 3c) | 4a) ★★ | 1c) | 1c) ★ | 2b) | 3 |
| Fathima et al (2018) | 1a) ★ | 2a) ★ | 3c) | 4b) ★ | 1c) | 1c) ★ | 2b) | 4 |
| Lazo-Porass et al (2023) | 1b) ★ | 2b) | 3c) | 4b) ★ | 1c) | 1c) ★ | 2b) | 3 |
| Liu et al (2021) | 1a)/b) ★ | 2b) | 3a) ★ | 4b) ★ | 1c) | 1c) ★ | 2b) | 4 |
| Chu et al (2021) | 1c) | 2b) | 3a) ★ | 4a) ★★ | 1c) | 1c) ★ | 2b) | 4 |
| Fowler (2012) | 1c) | 2b) | 3a) ★ | 4a) ★★ | 1c) | 1c) ★ | 2b) | 4 |
| Yehle et al (2012) | 1b) ★ | 2b) | 3c) | 4b) ★ | 1c) | 1c) ★ | 2b) | 3 |

a. Maximum of 10 stars can be achieved; maximum of one star per numbered item within all categories except for ascertainment of the exposure in the Selection category, Comparability and assessment of outcome in the Outcome category where a maximum of 2 stars can be achieved.

★ Criteria met

**Supplementary Table 8.** Modified Newcastle-Ottawa Scale (NOS) tool criteria for cross-sectional descriptive studies^a^

| **Selection** | | | | **Comparability** | **Outcome** | |
| --- | --- | --- | --- | --- | --- | --- |
| 1. Representativeness of the sample  a) Truly representative of the average individual ★ (all subjects or random sampling)  b) Somewhat representative of the average individual ★ (non-random sampling)  c) Selected group of users  d) No description of the sampling strategy | 2. Sample size  a) Justified and satisfactory ★  b) Not justified | 3. Non-respondents  a) Comparability between respondents and non-respondents characteristics is established and/or the response rate is satisfactory (≥80%) ★  b) The response rate is unsatisfactory, or the comparability between respondents and non-respondents is unsatisfactory  c) No description of the response rate or the characteristics of the responders and the non-responders | 4. Ascertainment of the exposure (risk factor)  a) Validated measurement tool ★★ (note study must specify validity)  b) Non-validated measurement tool, but the tool is available or described★  c) No description of the measurement tool | 1. The subjects in different outcome groups are comparable, based on the study design or analysis. Confounding factors are controlled.  a) The study controls for age ★  b) The study controls for sex ★  c) The study does not control for age or sex | 1 Assessment of outcome    a) Independent blind assessment ★★  b) Record linkage ★★  c) Self report ★  d) No description | 2. Statistical test  a) The statistical test used to analyze the data is clearly described and appropriate, and the measurement of the association is presented, including confidence intervals and/or probability level (p value) ★  b) The statistical test is not appropriate, not described, incomplete or not completed |

a. Studies must be allocated one rating per numbered item with the exception of up to two ratings (‘a’ and ‘b’) under ‘comparability’

★ Criteria met

**Supplementary Table 9.** Quality assessment of non-randomized evaluation studies using a modified Newcastle-Ottawa Scale (NOS) tool

|  | **Selection** | | | **Comparability** | **Outcome** | | | |  |
| --- | --- | --- | --- | --- | --- | --- | --- | --- | --- |
| **Article** | **1** | **2** | **3** | **1** | **1** | **2** | **3** | **4** | **Total stars^a^** |
| Crouch et al (2023) | 1c) | 2a) ★ | 3b) ★ | 1c) | 1c) ★ | 2b) | 3d) | 4a) ★ | 4 |
| Land et al (2016) | 1b) ★ | 2b) | 3b) ★ | 1c) | 1c) ★ | 2a) ★ | 3b) ★ | 4b) | 5 |
| Lloyd-Sherlock et al (2018) | 1c) | 2b) | 3b) ★ | 1c) | 1c) ★ | 2a) ★ | 3c) | 4b) | 3 |
| Hueston (1989) | 1c) | 2b) | 3b) ★ | 1c) | 1c) ★ | 2a) ★ | 3b) ★ | 4b) | 4 |
| Sinopoli & Lawless (2012) | 1b) ★ | 2b) | 3b) ★ | 1c) | 1c) ★ | 2b) | 3d) | 4b) | 3 |
| Sopko et al (1977) | 1b) ★ | 2a) ★ | 3b) ★ | 1c) | 1c) ★ | 2b) | 3d) | 4a) ★ | 5 |

a. Maximum of 11 stars can be achieved; maximum of one star per numbered item within all categories except for ascertainment of the exposure in the Selection category, Comparability and assessment of outcome in the Outcome category where a maximum of 2 stars can be achieved.

★ Criteria met

**Supplementary Table 10.** Modified Newcastle-Ottawa Scale (NOS) tool criteria for non-randomized evaluation studies^a^

| **Selection** | | | **Comparability** | **Outcome** | | | |
| --- | --- | --- | --- | --- | --- | --- | --- |
| 1.Representativeness of the exposed cohort  a) truly representative of the average individual ★  b) somewhat representative of the average individual ★  c) selected group of users  d) no description of the derivation of the cohort | 2. Sample size  a) Justified and satisfactory ★  b) Not justified | 3. Ascertainment of exposure  a) Validated measurement tool ★★ (note study must specify validity or pilot testing)  b) Non-validated measurement tool, but the tool is available or described★  c) No description of the measurement tool | 1. Comparability of cohorts on the basis of the design or analysis  a) The study controls for age ★  b) The study controls for sex ★  c) The study does not control for age or sex | 1. Assessment of outcome  a) Independent blind assessment ★★  b) Record linkage ★★  c) Self report ★  d) No description | 2. Was follow-up long enough for outcomes to occur^2^  a) yes (≥1 month) ★  b) no | 3. Adequacy of follow up of cohorts^b^  a) Complete follow up - all subjects accounted for ★  b) Subjects lost to follow up unlikely to introduce bias - small number lost - ≥80% follow up, or description provided of those lost) ★  c) Follow up rate <80% and no description of those lost  d) No statement | 4. Statistical test  a) The statistical test used to analyze the data is clearly described and appropriate, and the measurement of the association is presented, including confidence intervals and/or probability level (p value) ★  b) The statistical test is not appropriate, not described, incomplete or not completed |

a. Studies must be allocated one rating per numbered item with the exception of up to two ratings (‘a’ and ‘b’) under ‘comparability’

b. Only relevant for outcomes assessed during/after the intervention (e.g. attitudes)

★ Criteria met

**Supplementary Table 11. Quality assessment of randomized evaluation studies using the Cochrane Risk of Bias (ROB) 2.0 tool**

| **Article** | **Domain 1** | **Domain 2** | **Domain 3** | **Domain 4** | **Domain 5** | **Total risk of bias^a^** |
| --- | --- | --- | --- | --- | --- | --- |
| Barros et al (2015) | High risk | Some concerns | Low risk | Some concerns | Some concerns | High risk of bias |
| Charlton et al (2008) | Some concerns | Low risk | Low risk | Low risk | Some concerns | Some concerns |
| Eyles et al (2023) | Low risk | Low risk | Low risk | Some concerns | Some concerns | Some concerns |
| Li et al (2009) | Low risk | Low risk | Low risk | Low risk | Some concerns | Some concerns |
| Maleki et al (2016) | Some concerns | Some concerns | Low risk | Low risk | Some concerns | High risk of bias |
| Pan et al (2017) | Low risk | Low risk | Some concerns | Some concerns | Some concerns | High risk of bias |
| Pietinen et al (1981) | High risk | Some concerns | Low risk | Low risk | Some concerns | High risk of bias |
| Wang et al (2016) | Low risk | Some concerns | Low risk | Some concerns | Some concerns | Some concerns |
| Yu et al (2021) | Low risk | Low risk | Low risk | Low risk | Low risk | Low risk of bias |

1. Total risk of bias was determined high where studies scored “some concerns” in ≥3 or more domains
